# Regulatory aspects of optogenetic research and therapy for retinitis pigmentosa under EU law

**DOI:** 10.3389/fmedt.2025.1548927

**Published:** 2025-05-09

**Authors:** Johannes Freise

**Affiliations:** Law School, Faculty of Law, Economics and Business, Martin Luther University Halle-Wittenberg, Halle (Saale), Germany

**Keywords:** law, optogenetics, retinitis pigmentosa, rare diseases, combined studies, regulation of medicinal products, orphan medicinal products, regulation of medical devices

## Abstract

Optogenetics has potentials for a treatment of retinitis pigmentosa and other rare degenerative retinal diseases. The technology allows controlling cell activity through combining genetic engineering and optical stimulation with light. First clinical studies are already being conducted, whereby the vision of participating patients who were blinded by retinitis pigmentosa was partially recovered. In view of the ongoing translational process, this paper examines regulatory aspects of preclinical and clinical research as well as a therapeutic application of optogenetics in ophthalmology. There is no prohibition or specific regulation of optogenetic methods in the European Union. Regarding preclinical research, legal issues related to animal research and stem cell research have importance. In clinical research and therapeutic applications, aspects of subjects' and patients' autonomy are relevant. Because at EU level, so far, no specific regulation exists for clinical studies in which a medicinal product and a medical device are evaluated simultaneously (combined studies) the requirements for clinical trials with medicinal products as well as those for clinical investigations on medical devices apply. This raises unresolved legal issues and is the case for optogenetic clinical studies, when for the gene transfer a viral vector classified as gene therapy medicinal product (GTMP) and for the light stimulation a device qualified as medical device are tested simultaneously. Medicinal products for optogenetic therapies of retinitis pigmentosa fulfill requirements for designation as orphan medicinal product, which goes along with regulatory and financial incentives. However, equivalent regulation does not exist for medical devices for rare diseases.

## 1 Introduction

In 2021, a study was published in which the vision of a person blinded by retinitis pigmentosa was partially recovered using optogenetics (1). Optogenetics is a technology for controlling and monitoring the activity of cells (2–4). It is based on a combination of genetic engineering and optical light stimulation. Via gene transfer foreign genes are introduced into the target cells, which cause the expression of light-sensitive ion channels. In this way, by using photoreceptor sequences from microalgae, bacteria or fungi the cells become light-sensitive, and their activity can be controlled by light stimulation (4). While most optogenetic concepts are based on stimulation with light of a specific wavelength through an optical device, a recently published case report demonstrated an approach to optogenetic treatment using a synthetic opsin (synthopsin) that causes the targeted human retinal cells to become responsive to ambient light (5).

Besides the use of optogenetics as an instrument in basic research in animal models or *in vitro* models, research is being conducted on various therapeutic approaches. Objects of research are for example, the development of optogenetic cochlear implants (6), treatment options for neurodegenerative diseases such as Parkinson (7, 8) and Alzheimer's disease (9) as well as Retinitis Pigmentosa (1, 5, 10). Clinical studies are so far only performed concerning degenerative retinal diseases. In 8 of 11 studies registered on ClinicalTrials.gov, optogenetic techniques are applied in humans to investigate optogenetic therapeutic approaches for retinitis pigmentosa^1^. On the one hand, the ongoing studies underline therapeutic potentials of optogenetics in ophthalmology (11–13), on the other, they demonstrate the need for legal [and ethical (14)] analysis. Early recognition of potential medico-ethical and regulatory challenges and working on regulatory approaches can promote the clinical translation. Nevertheless, it is necessary to balance opportunities and risks (15). Considering this, the review examines regulatory implications of the translation process of optogenetics for a treatment of retinitis pigmentosa, taking ethical aspects into account and focusing on the law of the European Union (EU). Since retinitis pigmentosa has a prevalence of 1:4000 (16) it is classified as rare disease (17). By the EU rare diseases are defined as those with a prevalence of less than 5 per 10,000 people within the Union (18). By analysing EU's legislation on medicinal products and medical devices, regulatory challenges are to be identified, and initial approaches will be discussed. The review is conducted following a scoping approach. Its aim is to provide an overview on relevant legal aspects and thereby a basis for further interdisciplinary discussion on optogenetics.

## 2 Preclinical research

If preclinical research on the treatment of retinitis pigmentosa with optogenetics is performed in animal models (10, 19–21), aspects of animal testing regulations are of relevance, for the use of *in vitro* models (22–24) regulatory issues concerning the use of stem cells.

The law on animal experiments is largely harmonized in the EU by Directive 2010/63/EU on the protection of animals used for scientific purposes. In accordance with Art. 288 TFEU, the directive must be implemented into national law by the Member States. The Member States have to ensure that animal experiments are only carried out after prior official project evaluation by a national component authority (Art. 36 Directive 2010/63/EU). There are no special regulatory requirements for optogenetic research in animal models and the general provisions for animal experiments apply. For instance, animals with a higher sensitivity enjoy greater protection under Art. 13(2)(b) Directive 2010/63/EU. Besides other provisions, in Article 4 Directive 2010/63/EU the 3Rs principle (replacement, reduction, refinement) developed by Burch and Russel (25) is laid down in EU Law (26). The principle of replacement can be found in Art. 4(1) Directive 2010/63/EU and prohibits animal experiments if the use of a scientifically satisfactory alternative method is possible.

In ophthalmological research, *in vitro* models, for example using human retinal organoids, can be such an alternative method (6, 22–24). Organoids are three-dimensional cell cultures derived from embryonic stem cells (ESCs), induced pluripotent stem cells (iPSCs) or adult stem cells (ASCs) which model structures and functions of tissue (27). Due to their similarities to the human retina, human retinal Organoids can also open further research opportunities (28–30). From a legal perspective their use has its own implications. However, the bioethical discourse on consciousness and the moral and legal status of human cerebral organoids (31–34) has no greater relevance for the use of retinal organoids. Therefore, the ethical and legal evaluation of research with retinal organoids is less controversial, but by no means trivial.

Depending on the used stem cell type, the generation of organoids is subject to specific legal requirements that are not harmonized within the EU. The use of ESCs for research purposes is regulated very differently worldwide, an example of restrictive regulation is given by Germany (35). This legislation creates various legal obstacles for generating of and research with organoids derived from embryonic stem cells.

In contrast, the use of iPSCs or ASCs (including for the derivation of organoids) is not regulated by equivalent legislation. Nevertheless, questions regarding the interests of cell donors must be addressed. With regard to the requirements for the scope and specificity of information and consent, various consent models are discussed, for stem cell research in general and particularly in the context of organoids (36). If organoids are generated from stem cells, this should be part of the information, but it is not legally required to provide information about specific characteristics of (retinal) organoids (37). Considering biotechnological advances, governance models (38–40) and their potential legal implementation must be discussed further.

There are overlaps between regulatory aspects of animal experiments and stem cell research when human organoids are transplanted into animals. Retinal organoids also promise potentials for transplantation therapies, including for the treatment of retinitis pigmentosa (41). While the transplantation and integration of human cerebral organoids into non-human animals' brains is raising a discussion about their “moral-humanization” (42–44), this is not the case for a transfer of retinal organoids, because this does not involve an integration of human brain cells into the host's brain. There are no specific implications for such research that go beyond general aspects of animal experimentation regulation for invasive procedures and stem cell law.

## 3 Clinical research

### 3.1 Combined studies

In the registered studies, such as those by Sahel et al. and by Mohanty et al., the gene transfer is induced by a viral vector^2^ (1, 5). Optogenetic viral vectors encoding a light-sensing opsin for optogenetic therapies classify as gene therapy medicinal products (GTMP) as defined in Annex I, Part IV, 2.1 Directive 2001/83/EC (45). This applies whether stimulation by an optical device is required for potentially therapeutic effects. According to Art. 2(1)(a) Regulation (EC) No 1394/2007 (ATMP Regulation) GTMPs are advanced therapy medicinal products (ATMPs).

Insofar as the optogenetic stimulation serves medical purposes, optical devices [in ophthalmology special goggles (1) or headsets (46)] used for light simulation qualify as medical devices within the meaning of Art. 2 No. 1 Regulation (EU) No 2017/745 (MDR) (15, 47).

With exception of the synthetic opsin-based approach, optogenetic GTMPs and optical medical devices only achieve a potential therapeutic effect through an interplay. This means that testing therapeutic effects in humans and also for a safety evaluation, how the genetically altered neurons react to light stimulation, a simultaneous evaluation is necessary (45). Those clinical studies, in which both a medical device and a separate medicinal product are evaluated are called combined studies (48). However, the legal framework for such studies is complex (45).

On the EU level there is no specific regulation for combined studies. Specific regulation exists only regarding combination products, in which an ATMP has a medical device as an integral part (Art. 9 ATMP Regulation), a medical device with a medicinal product as an integral part [Art. 1(8) MDR] and for application devices [Art. 1(9) MDR]. However, in current optogenetic approaches, the GTMP and the optical medical device do not form an integral product. For combined studies the requirements for clinical trials with medicinal products and for clinical investigations of medical devices apply in parallel (48). The Regulation (EU) No 536/2014 (CTR) governs only clinical trials of medicinal products for human use, whereas clinical investigations on medical devices are largely regulated by Art. 62–80 MDR. As the provisions are not synchronized regulatory issues occur planning and conducting combined studies (48).^3^

Before conducting a combined study, it is necessary to apply for two authorizations, one in accordance with the requirements of the CTR and one under the MDR. In some Member States, it is not the same authority competent for both applications. Also, for the ethical evaluation as both the CTR as well as the MDR require, in some states two separate applications are necessary and different ethics committees responsible. This not only creates administrative burdens (48), but it also raises the legal question of the consequences of differing assessments results. One of the challenges for conducting combined studies is that the requirements for reporting safety events are not harmonized between CTR and MDR (48).

Even if the use of synthetic opsins could become standard for optogenetic treatments in ophthalmology, in other fields of application the use of optical medical devices would still be essential. This is the case for specific control of target cells for optogenetic hearing restauration with optical cochlea implants (6) as well as for sufficient light stimulation in the brain (4). At least in these fields, combined studies remain necessary, and the associated regulatory challenges would persist.

### 3.2 Participant autonomy

Clinical research with individuals capable of giving consent may only be conducted if they have given their informed consent. As a guiding ethical principle this is enshrined in the Helsinki Declaration (49). This principle is also legally binding by various national, European and international legal documents. The CTR (Articles 28–35) and the MDR (Articles 63–68) also include specific provisions to protect subjects' autonomy. CTR and MDR contain different regulation for investigations on minors and incapacitated subjects, which is a further challenge for combined studies. While this does not seem to be of greater significance regarding retinitis pigmentosa, future optogenetic studies on neurodegenerative diseases such as Alzheimer's could also include subjects who are incapable of giving consent.

Like in other studies on gene therapies, in optogenetic trials, it must be taken into account that the irreversibility of the genetic modification may cause subjects to no longer be eligible for other future treatment methods (14). At the same time, due to the current lack of treatment options for rare diseases like retinitis pigmentosa, on the one hand, participating in a trial gives patients hope for a therapy, on the other, it can make them accept more risks and thus particularly vulnerable (50). To provide legally compliant information, it must be ensured that false expectations of the subjects, regarding a therapeutic benefit are prevented [therapeutic misconception (51)] and short- and long-term risks of participating in an optogenetic trial are made clear. This is also essential for ethical reasons (14). It is especially important when it comes to participants who also suffer psychologically from their condition. In case of retinitis pigmentosa and other degenerative retinal diseases, vision loss can cause psychological strain and anxiety (52).

Moreover, as in other clinical research projects, the emergence of misconceptions regarding the social value of participation must be avoided (social value misconception) (53).

If the information is not done adequately, the consent based on it may be legally invalid. Should a physical intervention be carried out despite ineffective consent, for example under German law scientists and physicians risk criminal prosecution for bodily harm.

## 4 Regulation of medicinal products

As an ATMP the GTMP component of optogenetic therapies may only be placed on the market in the EU if this has been authorized in accordance with Regulation (EC) No 726/2004. For ATMPs the centralized European procedure applies [Art. 3(1) in conjunction with Annex I No. 1a Regulation (EC) No 726/2004]. An application for market authorization requires, among other obligations, successful clinical trials [Art. 6(1) Regulation (EC) No 726/2004]. An exception from the centralized procedure applies to ATMPs manufactured on a non-routine basis in a hospital (Art. 3 No. 7 Directive 2001/83/EC), for those national procedures apply.

Due to retinitis pigmentosa classifies as rare disease, a designation of optogenetic GTMPs as orphan medicinal products according to Regulation (EC) No 141/2000 may be considered. As retinitis pigmentosa affects no more than 5 in 10,000 people in the EU and results in chronic disability, the requirements of the regulation are met. The designation does not take place automatically. Instead, according to Art. 5(1) Regulation (EC) No 141/2000, for the designation an application to the European Medicines Agency (EMA) is required. Following examination by the EMA's Committee for Orphan Medicinal Products (COMP), the outcomes are forwarded to the European Commission, which is competent for granting EU's orphan status. An application can be made at any time during development and therefore already based on preclinical data (54). The optogenetic vector GS030-Drug Product (GS030-DP) evaluated in the study of Sahel et al. received the orphan designation by the European Commission in 2017.

For all designated orphan medicinal products, the centralized European marketing authorization procedure applies [Art. 3(1) in conjunction with Annex I No. 4 Regulation (EC) No 726/2004]. Since the centralized procedure is, in principle, mandatory for ATMPs, this has no further significance for optogenetic GTMPs. Nevertheless, the recognition grants regulatory and financial privileges that should provide incentives for corresponding research [Art. 1 Regulation (EC) No 141/2000]. The limited number of patients poses a challenge for the design of studies on rare diseases like retinitis pigmentosa (5). Incentives should enable a better supply of medicinal products for patients with rare diseases. Sponsors receive protocol assistance and are exempted from fees. After an orphan medicinal product receives marketing authorization, in principle, 10 years of market exclusivity over similar medicinal products for the same indication are granted. However, granting market exclusivity can also have negative impacts on other research (55). But as no optogenetic therapy has yet been authorized, currently no market exclusivity rights apply in this field.

To treat retinitis pigmentosa, another (non-optogenetic) GTMP (Luxturna) designated as an orphan medicinal product was already authorized in November 2018. Its use is limited to patients with confirmed biallelic RPE65 mutations with enough functioning retinal cells and decelerates the diseases progression (56). Optogenetic treatment approaches are not limited to defects of the RPE65 gene and are intended for patients at a later stage of the disease (13). Consequently, GTMPs for optogenetic therapy would not conflict with the granted market exclusivity.

## 5 Regulation of medical devices

As medical devices, special goggles (1) or headsets (46) for optogenetic stimulation to treat Retinitis pigmentosa may only be placed on the market or put into service if they fulfill the requirements of the MDR when properly supplied, correctly installed and maintained and used in accordance with their intended purpose [Art. 5(1) MDR]. According to Art. 52 MDR manufactures are obliged to undertake a conformity assessment procedure. Medical devices which comply the legal requirements are labelled with the CE mark.

The type and scope of the proof of safety and performance requirements vary on the risk class. The MDR categorizes medical devices into four different risk classes (I, IIa, IIb, III). Unlike an application of optogenetics in the brain or the cochlea would, optical medical devices for retinitis pigmentosa do not require an implantation. Nevertheless, because the devices are intended to deliver energy to the body they classify as an active therapeutic device, depending on the potential risks assigned to class IIa or IIb according to VIII Chapter III Rule 9 MDR (47). For classification, the sensitivity of the eye on the one hand and the fact that the energy supplied is light energy on the other must be considered. However, the risks cannot be conclusively determined in this paper. Whereas optical cochlear implants as active implantable medical devices fall minimum into risk class IIb. Due to the direct contact to the central nervous system, optogenetic brain implants would classify as class III devices (15, 47).

In contrast to orphan medicinal products, in the EU no regulatory privileges for medical devices for rare diseases exist. The guidelines for orphan medicinal devices recently published by the EU's Medical Device Coordination Group (MDCG) (57) do not change this. Instead, the guidelines merely set out the regulations contained in the MDR in respect of orphan devices. Furthermore, without apparent reasons, they are based on a narrower understanding of orphan status and define orphan devices as medical devices that are used to treat, diagnose or prevent a disease or condition affecting no more than 12,000 people in the EU (57).

Calls for special regulation of orphan devices (58) have been taken up by the European Parliament recently (59). Manufacturers can be faced with high development and production costs on the one hand and relatively low sales potential on the other (58). To improve the supply of medical devices for rare diseases, specific legal and financial incentives for manufacturers would be favorable. Whether the European Commission will comply with this and present a corresponding draft law remains to be seen. Any implementation should incorporate a critical perspective on market exclusivity rights, as they exist for orphan medicinal products.

## 6 Therapeutic application

As in clinical research, respect for the autonomy of the person being treated is central in any future therapeutic application of optogenetics. In principle, informed consent for medical interventions requires a comprehensive explanation of the risks and effects of the treatment. However, the complexity of some (emerging) methods raises the question of whether comprehensive and understandable information can be provided to patients (60). This would also apply to optogenetic therapies.

In the future, legal questions regarding the coverage of optogenetic therapies within public healthcare systems will also need to be clarified. Not only in the case of optogenetics, rare diseases like retinitis pigmentosa go along with legal issues of social security (61, 62). In the EU, this also raises questions of cross-border healthcare (62), which is regulated by Directive 2011/24/EU.

## 7 Conclusion

Despite the existing regulatory challenges, there are no legal obstacles that would prevent the clinical translation of optogenetic therapies for retinitis pigmentosa and other rare degenerative retinal diseases. Nevertheless, particularly regarding the regulation of combined studies amendments are necessary. Although some member states already established coordinated assessments at national level, the implementation of a single authorization procedure for combined studies under EU law is still required. Even if in ophthalmology synthetic opsin-based approaches without the use of optical medical devices could be established, amendments would facilitate future optogenetic studies on optical cochlear implants or the treatment of neurodegenerative diseases. The opaque legal framework is a hurdle to research that should be overcome.

Additionally, aspects of subject and patient autonomy in connection with complex medical technologies as well as rare diseases need to be discussed further.

## Footnotes

^1^ClinicalTrials.gov identifiers: NCT02556736, NCT03326336, NCT04919473, NCT04945772, NCT05294978, NCT05921162, NCT06162585, NCT06292650, NCT06460844 (status as of 22 April 2025).

^2^Sahel et al. used a serotype 2.7m8 adeno-associated viral vector encoding the light-sensing channelrhodopsin protein ChrimsonR fused to the red fluorescent protein tdTomato (1). In the study of Mohanty et al. for delivering the synthopsin, an AAV2 gene expression cassette vMCO-010 was generated (5).

^3^In light of this, the European Commission and the member states’ competent national authorities initiated the ‘COMBINE’ project, to analyze current challenges and work with other stakeholders on possible solutions (48). The project also includes the parallel challenges of evaluating a medicinal product and an in-vitro diagnostic product together. In-vitro diagnostics are not subject to the same requirements as other medical devices, instead to the specific provisions of the Regulation (EU) No 2017/746 (IVDR).
